# Supplementary material for: ASK1 inhibits browning of white adipose tissue in obesity
Source: Nat Commun. 2020 Apr 2;11:1642. doi: 10.1038/s41467-020-15483-7 (PMC7118089; doi:10.1038/s41467-020-15483-7)
Supplement: Supplementary file 1 — Supplementary Information [file 41467_2020_15483_MOESM1_ESM.pdf]

ASK1 inhibits browning of white adipose tissue in obesity

Fabrizio C. Lucchini, Stephan Wueest, Tenagne D. Challa, Flurin Item, Salvatore Modica, Marcela Borsigova, Yulia Haim, Christian Wolfrum, Assaf Rudich, Daniel Konrad

SUPPLEMENTARY INFORMATION

Supplementary Figures 1-6
Supplementary Table 1

Supplementary Figure 1

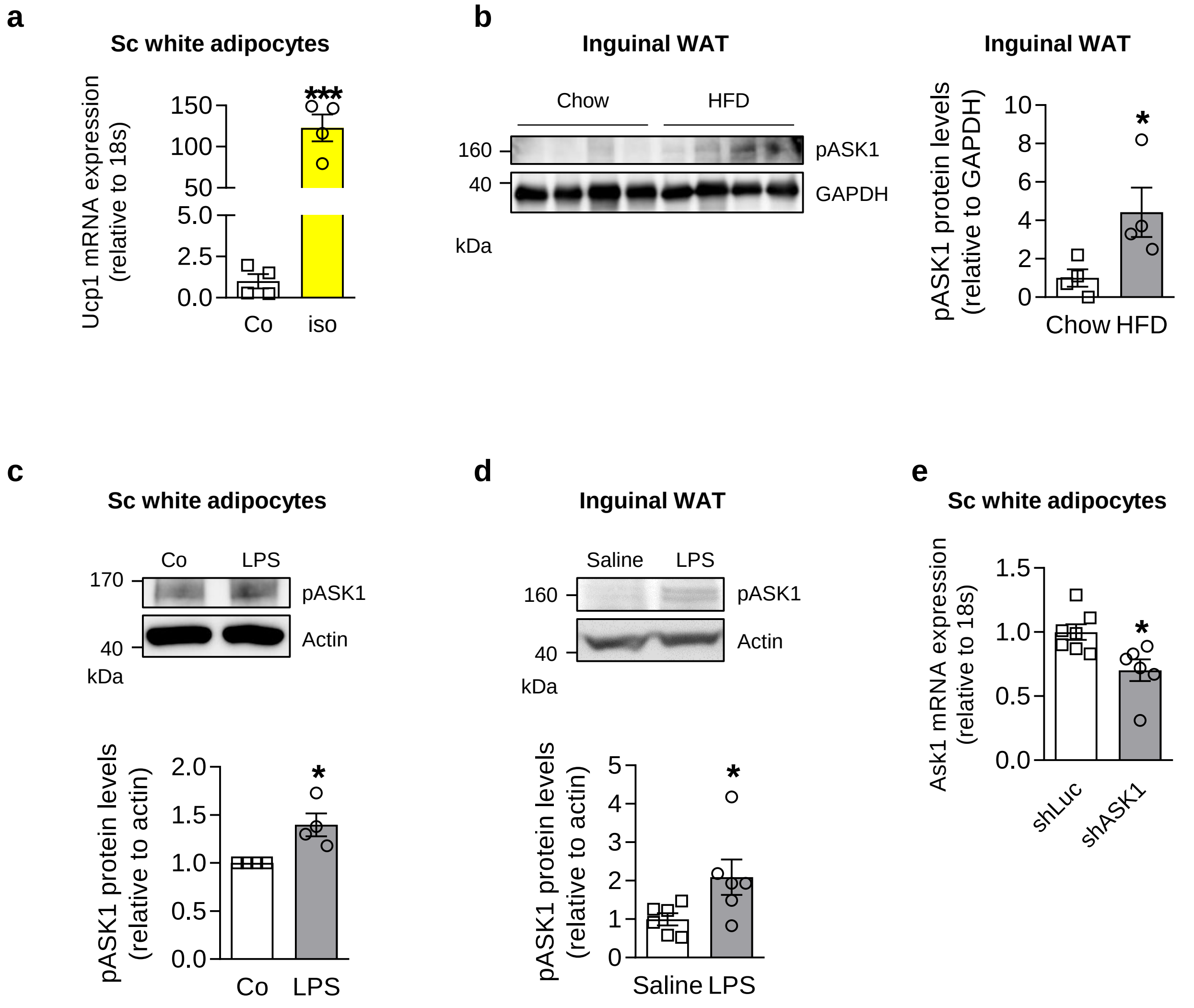

HFD and LPS increases pASK1 protein levels in white adipose tissue

(**a**) Ucp1 mRNA expression in subcutaneous white adipocytes treated with or without 0.1 µM isoproterenol for 6 hours (n=4 biological replicates). ***p=0.0003. (**b**) Western blot and quantified protein levels of pASK1 in total lysates of inguinal white adipose tissue harvested from C57BL/6J mice fed a chow or HFD for 20 weeks. n=4 (chow) and n=4 (HFD) mice per group. *p=0.046. (**c**) Western blot and quantified protein levels of pASK1 in total lysates of scWAT adipocytes treated with or without 100 ng/ml LPS for 1 hour. n=4 biological replicates. *p=0.044. (**d**) Western blot and quantified protein levels of pASK1 in total lysates of inguinal white adipose tissue harvested from cold-exposed C57BL/6J mice chronically treated with or without LPS using osmotic minipumps. n=6 mice per group. *p=0.049. (**e**) Ask1 mRNA expression in subcutaneous white adipocytes transfected with control shRNA lentivirus (shLuc; n=7 biological replicates) or shRNA lentivirus targeting Ask1 (shASK1; n=6 biological replicates). *p=0.014. Values are expressed as mean ± SEM. Statistical tests used: two-sided *t*-tests for **a**, **b**, **d** and **e**; two-sided one sample *t*-test for **c**. Source data are provided as a Source Data file.

Supplementary Figure 2

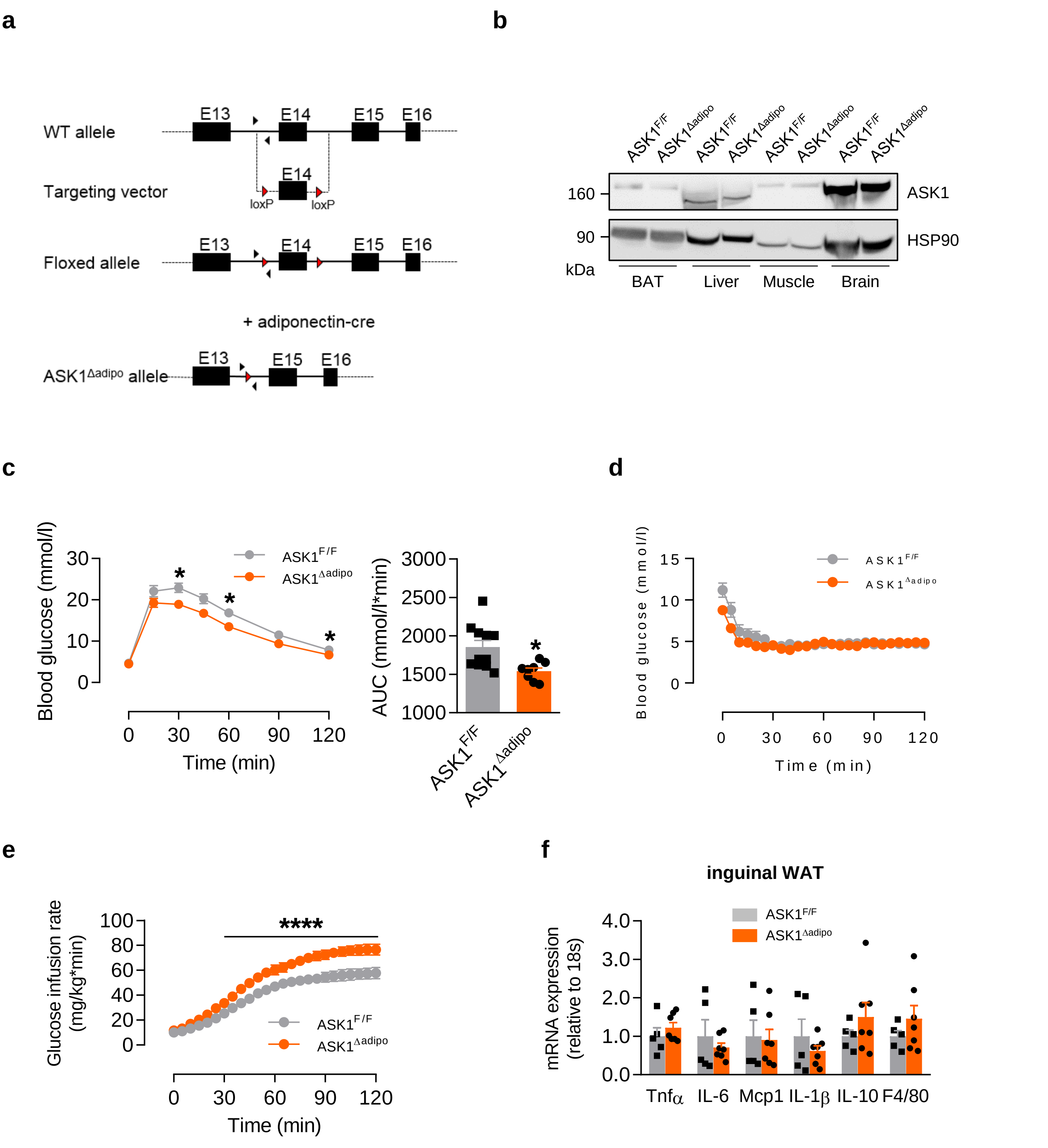

Generation of adipocyte-specific ASK1 knockout mice

(**a**) Schematic model of ASK1$^{\Delta adipo}$ generation. (**b**) Total tissue lysates were prepared from ASK1$^{F/F}$ and ASK1$^{\Delta adipo}$ mice (n=1 mouse per group) , resolved by LDS-PAGE and immunoblotted with anti-ASK1 or anti-HSP90 antibody. (**c**) Intraperitoneal glucose tolerance test (ASK1$^{F/F}$, n=11 mice; ASK1$^{\Delta adipo}$, n=8 mice) after 4 days of HFD-feeding. *p=0.049 (30 min), *p=0.036 (60 min), *p=0.023 (120 min), *p=0.010 (AUC). (**d**) Blood glucose levels were clamped during hyperinsulinemic-euglycemic clamp at about 5 mmol/l in HFD-fed ASK1$^{F/F}$ (n=6) and ASK1$^{\Delta adipo}$ (n=8) mice. (**e**) In order to maintain euglycemia, glucose infusion rate was adjusted over time. ****p<0.0001. (**f**) Inguinal adipose tissue mRNA expression of respective targets determined in HFD-fed ASK1$^{F/F}$ (n=5) and ASK1$^{\Delta adipo}$ (n=7) mice. Values are expressed as mean ± SEM. Statistical tests used: two-sided *t*-tests for **c** (AUC); ANOVA for **c**, **e**. Source data are provided as a Source Data file.

Supplementary Figure 3

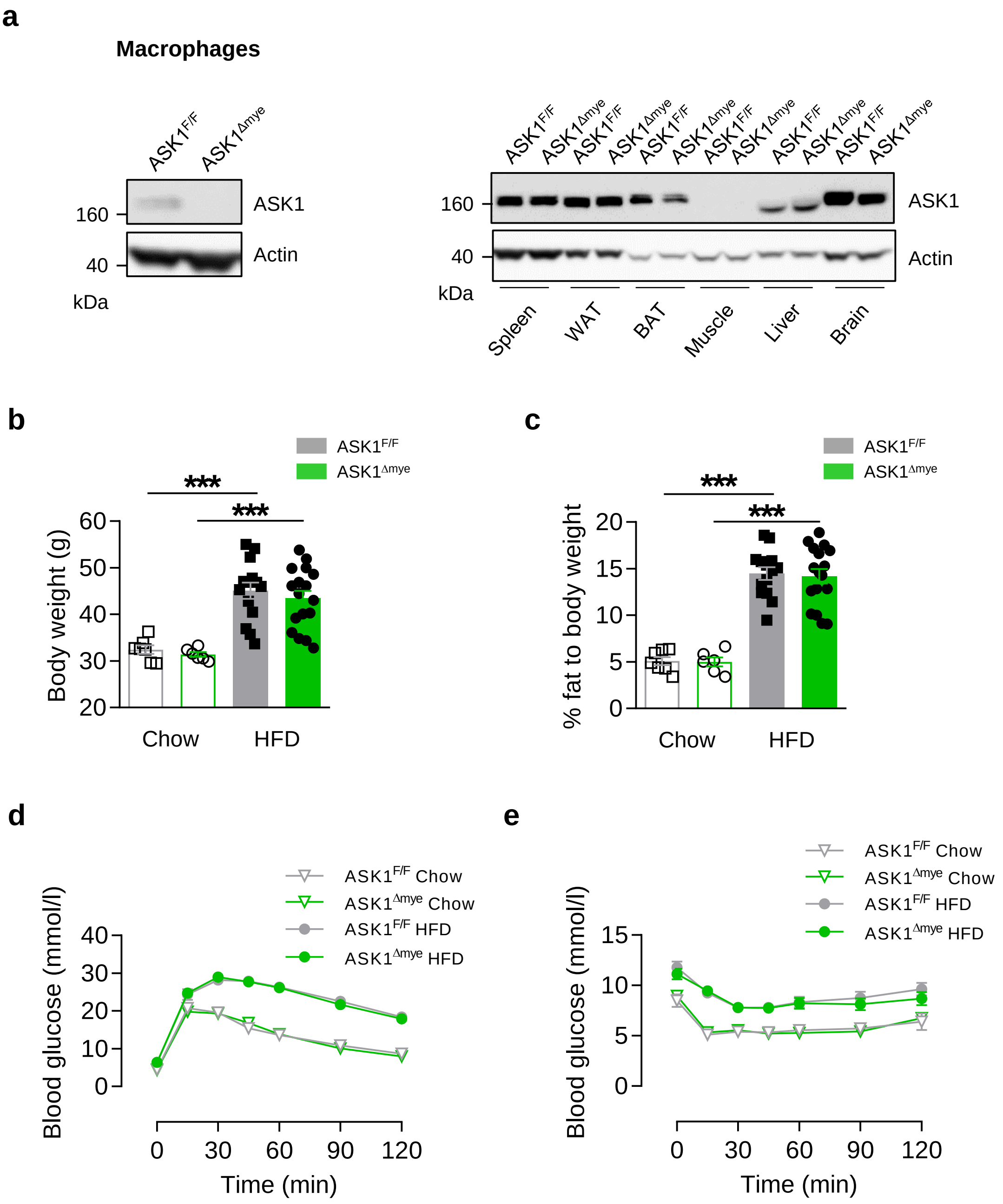

Characterization of myeloid-specific ASK1 knockout mice

(**a**) Protein levels of ASK1 in lysate of intraperitoneal macrophages or respective tissues harvested from ASK1$^{\Delta mye}$ and ASK1$^{F/F}$ mice (n=1 mouse per group). Lysates were resolved by LDS-PAGE and immunoblotted with anti-ASK1 or anti-actin antibody. (**b** and **c**) Body weight and body fat amount after 20 weeks of chow (ASK1$^{F/F}$, n=7; ASK1$^{\Delta mye}$, n=6) or HFD feeding (ASK1$^{F/F}$, n=14; ASK1$^{\Delta mye}$, n=17). ***p=0.0001 (for body weight ASK1$^{F/F}$), ***p=0.0004 (for body weight ASK1$^{\Delta mye}$), ***p<0.0001 (for % fat to body weight). (**d** and **e**) Intraperitoneal glucose and insulin tolerance test (chow-fed: ASK1$^{F/F}$, n=7; ASK1$^{\Delta mye}$, n=6; HFD-fed: ASK1$^{F/F}$, n=14; ASK1$^{\Delta mye}$, n=16 (ITT) or n=17 (GTT)) at 18 weeks of age. Values are expressed as mean ± SEM. Statistical tests used: ANOVA. Source data are provided as a Source Data file.

Supplementary Figure 4

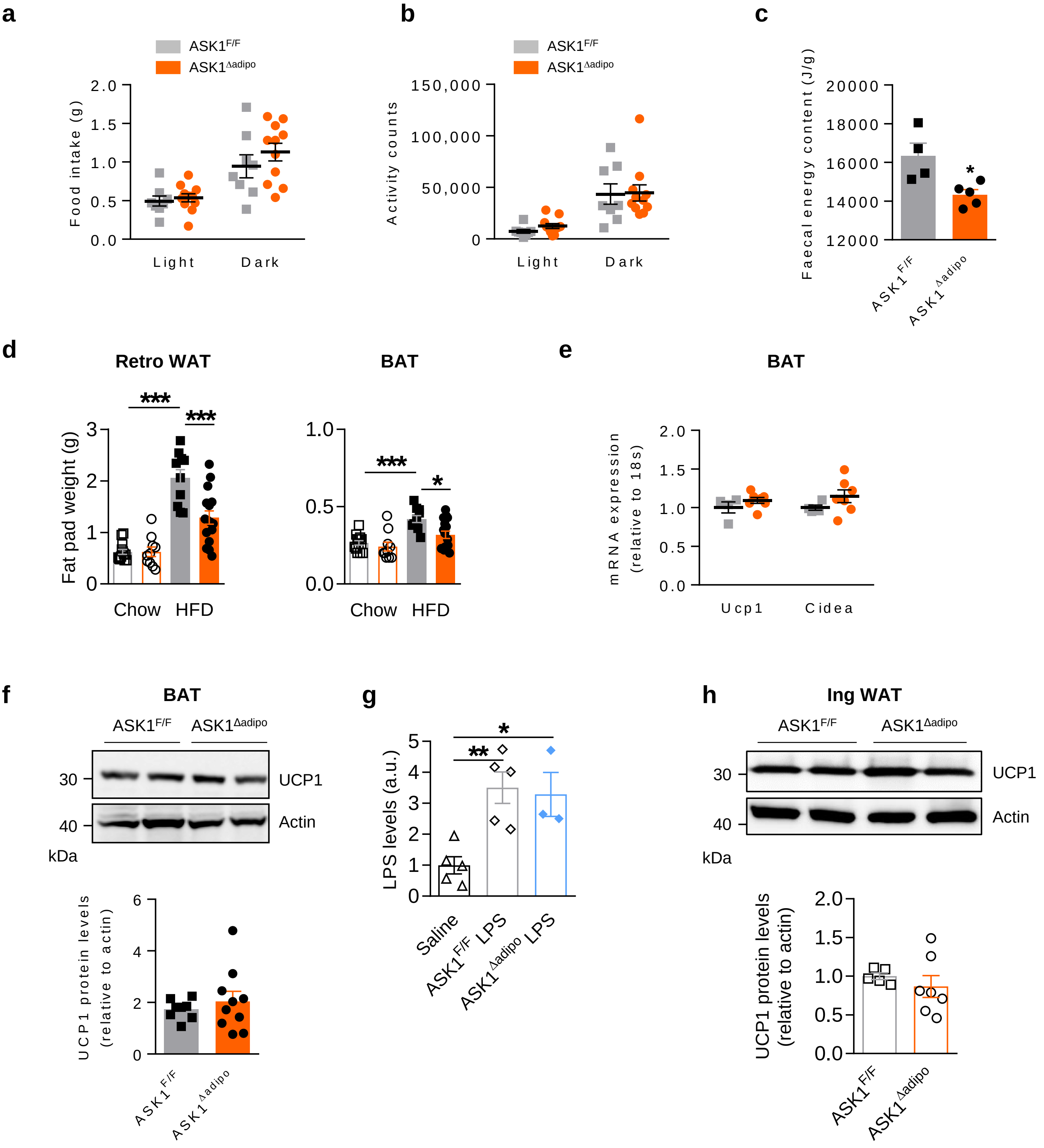

Characterization of ASK1$^{\Delta adipo}$ mice

Food intake (**a**) and locomotor activity (**b**) was determined in HFD-fed ASK1$^{F/F}$ (n=8) and ASK1$^{\Delta adipo}$ (n=11) mice during a 24-hour period at room temperature. (**c**) Faecal energy content was determined by bomb calorimetry determined in HFD-fed ASK1$^{F/F}$ (n=4) and ASK1$^{\Delta adipo}$ (n=5) mice. *p=0.019. (**d**) Fat pad mass of retroperitoneal white adipose tissue and BAT (chow-fed: ASK1$^{F/F}$, n=13; ASK1$^{\Delta adipo}$, n=10; HFD-fed: ASK1$^{F/F}$, n=10; ASK1$^{\Delta adipo}$, n=16) in 18 week old mice. *p=0.016, ***p=0.0002. (**e**) mRNA expression of respective genes in BAT of HFD-fed ASK1$^{F/F}$ (n=4) and ASK1$^{\Delta adipo}$ (n=7) mice. (**f**) Representative Western blot and quantification of UCP1 protein levels in BAT harvested from HFD-fed ASK1$^{F/F}$ (n=8) and ASK1$^{\Delta adipo}$ (n=10) mice. (**g**) Plasma LPS levels were determined in mice implanted with osmotic minipumps filled with saline (n=4) or LPS (n=5 ASK1$^{F/F}$ or n=3 ASK1$^{\Delta adipo}$). *p=0.025, **p=0.006. (**h**) Representative Western blot and quantification of UCP1 protein levels in inguinal adipose tissue harvested from cold-exposed chow-fed ASK1$^{F/F}$ (n=5) and ASK1$^{\Delta adipo}$ (n=7) mice. Values are expressed as mean ± SEM. Statistical tests used: two-sided *t*-tests for **c**; ANOVA for **d**, **g**. Source data are provided as a Source Data file.

Supplementary Figure 5

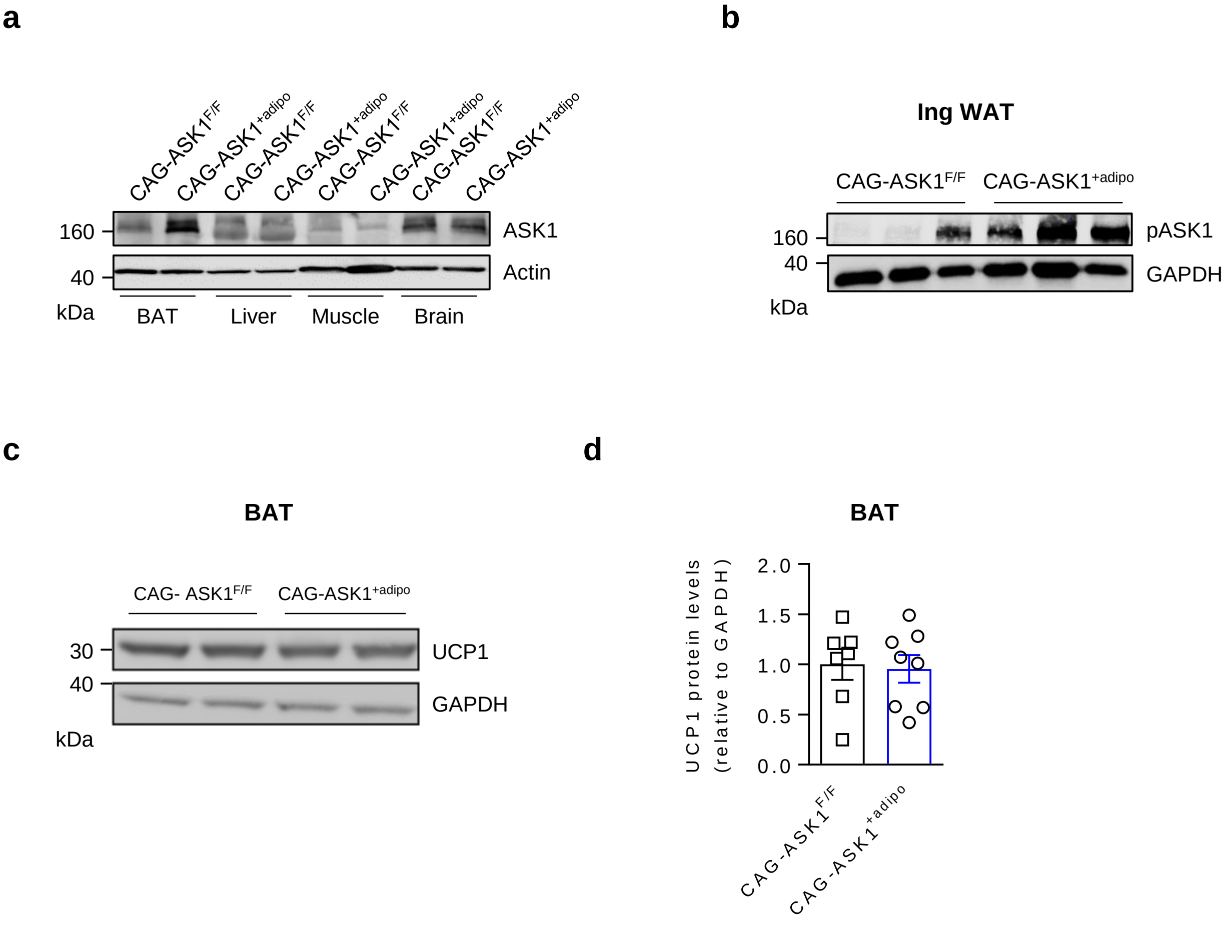

Characteristics of ASK1^{+adipo} mice

(**a**) Total tissue lysates were prepared from CAG-ASK1$^{F/F}$ and CAG-ASK1^{+adipo} mice (n=1 mouse per group), resolved by LDS-PAGE and immunoblotted with anti-ASK1 or anti-Actin antibody. (**b**) Western blot of pASK1 in total lysates of inguinal white adipose tissue harvested from cold-exposed CAG-ASK1$^{F/F}$ and CAG-ASK1^{+adipo} mice (n=3 mice per group) . Representative Western blot (**c**) and quantification (**d**) of UCP1 protein levels in BAT harvested from cold-exposed CAG-ASK1$^{F/F}$ (n=7) and CAG-ASK1^{+adipo} (n=8) mice. Values are expressed as mean ± SEM. Source data are provided as a Source Data file.

Supplementary Figure 6

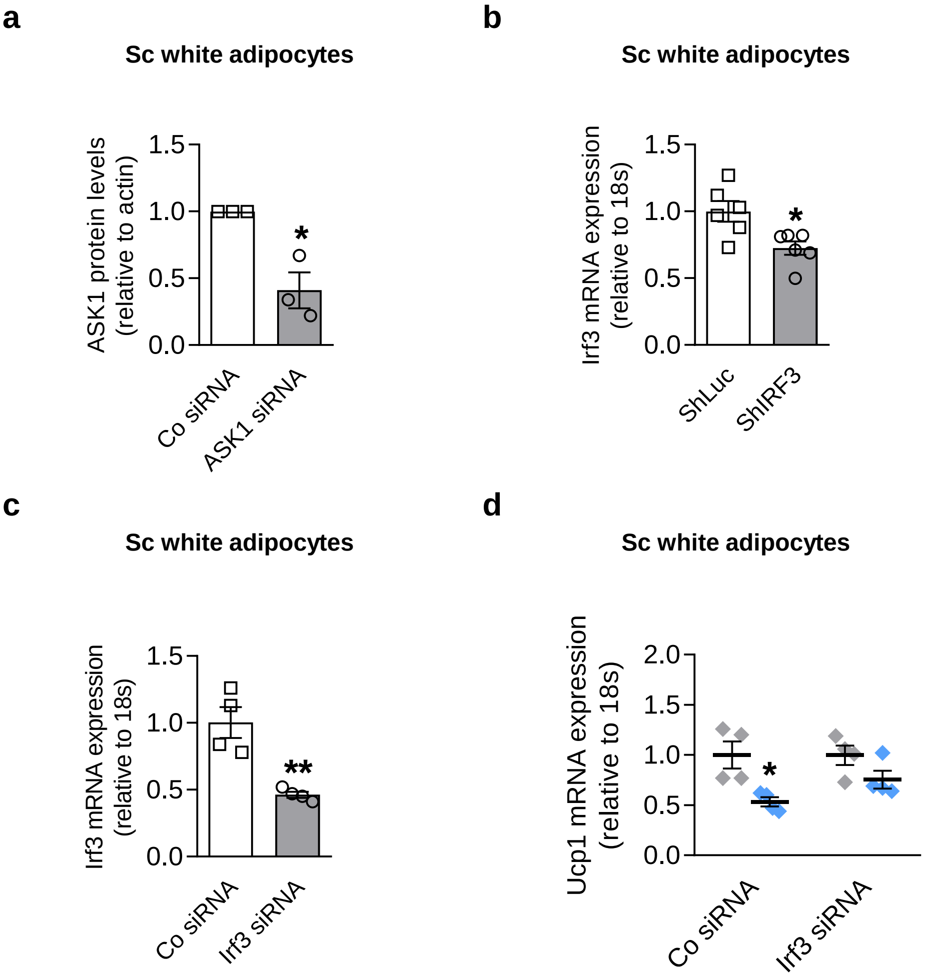

ASK1 and IRF3 knockdown in subcutaneous white adipocytes

(**a**) Quantification of ASK1 protein levels in subcutaneous white adipocytes transfected with control siRNA (Co siRNA) or siRNA targeting ASK1 (ASK1 siRNA) (n=3 biological replicates per group). *p=0.048. (**b**) Irf3 mRNA expression in subcutaneous white adipocytes transfected with control shRNA lentivirus (shLuc) or shRNA lentivirus targeting IRF3 (shIRF3) (n=6 biological replicates per group). *p=0.014. (**c**) Irf3 mRNA expression in subcutaneous white adipocytes transfected with control siRNA (Co siRNA) or siRNA targeting Irf3 (Irf3 siRNA) (n=4 biological replicates per group). **p=0.004. (**d**) Ucp1 mRNA expression in subcutaneous adipocytes transfected with control siRNA (Co siRNA) or siRNA targeting Irf3 (Irf3 siRNA) pre-treated with 100 ng/ml LPS for 24 hours followed by stimulation with 0.1 µM isoproterenol for 6 hours (n=4 biological replicates per group). *p=0.016. Values are expressed as mean ± SEM. Statistical tests used: two-sided one sample t test (a), two-sided t tests (b, c, d)). Source data are provided as a Source Data file.

Supplementary Table 1 Phenotypic characteristics of chow- and HFD-fed ASK1$^{F/F}$ and ASK1$^{\Delta adipo}$ mice

	ASK1$^{F/F}$ Chow	ASK1$^{\Delta adipo}$ Chow	ASK1$^{F/F}$ HFD	ASK1$^{\Delta adipo}$ HFD
Insulin (pmol/l)	n.d.	n.d.	3.1 ± 0.5 (n=6)	1.3 ± 0.3** (n=6)
Adiponectin (µg/ml)	67.0 ± 3.3 (n=4)	62.1 ± 3.1 (n=3)	47.2 ± 3.3# (n=7)	43.2 ± 2.3## (n=7)
TNFα (pg/ml)	11.5 ± 1.7 (n=4)	11.9 ± 0.5 (n=4)	12.1 ± 0.8 (n=10)	12.6 ± 0.9 (n=10)
IL-6 (pg/ml)	60.3 ± 15.4 (n=4)	55.6 ± 17.7 (n=4)	78.3 ± 28.3 (n=10)	95.9 ± 17.6 (n=10)
IL-10 (pg/ml)	18.6 ± 1.9 (n=4)	16.4 ± 0.9 (n=4)	21.4 ± 2.2 (n=10)	25.0 ± 2.5 (n=10)
IFN-γ (pg/ml)	0.39 ± 0.07 (n=4)	0.55 ± 0.13 (n=4)	0.59 ± 0.10 (n=10)	0.75 ± 0.14 (n=10)
KC (pg/ml)	52.3 ± 12.8 (n=4)	76.1 ± 12.9 (n=4)	87.0 ± 18.3 (n=10)	106.6 ± 11.0 (n=10)

Related to Figure 2. Mice were fasted for 5 hours before blood sampling. Values are expressed as mean ± SEM. #p=0.002 and ##p=0.007 indicate significant differences between diets of the same genotype (ANOVA), **p=0.007 indicate significant differences between genotypes of the same diet (Student's *t* test).
