# Supplementary material for: ASK1 inhibits browning of white adipose tissue in obesity
Source: Nat Commun. 2020 Apr 2;11:1642. doi: 10.1038/s41467-020-15483-7 (PMC7118089; doi:10.1038/s41467-020-15483-7)
Supplement: Supplementary file 3 — Reporting Summary [file 41467_2020_15483_MOESM3_ESM.pdf]

nature research

Corresponding author(s): Daniel Konrad

Last updated by author(s): Feb 4, 2020

Reporting Summary

Nature Research wishes to improve the reproducibility of the work that we publish. This form provides structure for consistency and transparency in reporting. For further information on Nature Research policies, see Authors & Referees and the Editorial Policy Checklist .

Statistics

For all statistical analyses, confirm that the following items are present in the figure legend, table legend, main text, or Methods section.

n/a	Confirmed	
☐	☒	The exact sample size (n) for each experimental group/condition, given as a discrete number and unit of measurement
☐	☒	A statement on whether measurements were taken from distinct samples or whether the same sample was measured repeatedly
☐	☒	The statistical test(s) used AND whether they are one- or two-sided<br>*Only common tests should be described solely by name; describe more complex techniques in the Methods section.*
☒	☐	A description of all covariates tested
☐	☒	A description of any assumptions or corrections, such as tests of normality and adjustment for multiple comparisons
☐	☒	A full description of the statistical parameters including central tendency (e.g. means) or other basic estimates (e.g. regression coefficient) AND variation (e.g. standard deviation) or associated estimates of uncertainty (e.g. confidence intervals)
☐	☒	For null hypothesis testing, the test statistic (e.g. F, t, r) with confidence intervals, effect sizes, degrees of freedom and P value noted<br>*Give P values as exact values whenever suitable.*
☒	☐	For Bayesian analysis, information on the choice of priors and Markov chain Monte Carlo settings
☒	☐	For hierarchical and complex designs, identification of the appropriate level for tests and full reporting of outcomes
☒	☐	Estimates of effect sizes (e.g. Cohen's d, Pearson's r), indicating how they were calculated

Our web collection on statistics for biologists contains articles on many of the points above.

Software and code

Policy information about availability of computer code

Data collection	TSE PhenoMaster software (TSE Systems, Bad Homburg, Germany)<br>siDESIGN center tool (Dharmacon)
Data analysis	GraphPad Prism 8.0.0. (GraphPad Software, San Diego, CA, USA)<br>Image Lab 5.2.1. (BioRad, Reinach, Switzerland)<br>CFX Maestro (BioRad, Reinach, Switzerland)

For manuscripts utilizing custom algorithms or software that are central to the research but not yet described in published literature, software must be made available to editors/reviewers. We strongly encourage code deposition in a community repository (e.g. GitHub). See the Nature Research guidelines for submitting code & software for further information.

Data

Policy information about availability of data

All manuscripts must include a data availability statement. This statement should provide the following information, where applicable:

- Accession codes, unique identifiers, or web links for publicly available datasets
- A list of figures that have associated raw data
- A description of any restrictions on data availability

Source data where each figure in the main manuscript and in the Supplementary Information is represented by a single sheet in an Excel document labelled “Source Data” is provided.

Field-specific reporting

Please select the one below that is the best fit for your research. If you are not sure, read the appropriate sections before making your selection.

[x] Life sciences [ ] Behavioural & social sciences [ ] Ecological, evolutionary & environmental sciences

For a reference copy of the document with all sections, see nature.com/documents/nr-reporting-summary-flat.pdf

Life sciences study design

All studies must disclose on these points even when the disclosure is negative.

Sample size	Sample size was determined based on previous experiments performed in our laboratory (EMBO Mol Med 2019; 11(10):e10124; Nat Commun 2017; 8(1):480; J Clin Invest 2010; 120(1):191-202).
Data exclusions	Data differing more than +/- 2 SD from the mean were excluded. This criteria was preestablished. Outliers were removed as they may not be part of the study population (i.e. unusual properties or conditions).
Replication	All data shown were successfully repeated in at least two independent experiments except for experiments presented in Figure 5e and Supplementary Figure 6d. Each experiment was performed multiple times as indicated in the figure legends.
Randomization	Not applicable for genetically modified mice as they were allocated based on their genotype. For experiments comparing wildtype chow vs. HFD-fed mice, animals were allocated to groups based on body weight (similar mean +/- SD in starting body weight between the groups). Age and sex was the same between the groups.
Blinding	For experiments comparing wildtype chow vs. HFD-fed mice, experimenters were not blinded to group allocations as colour of diet differed and hence, group allocation can be easily identified. For genetically modified mice, experimenters were mainly blinded to the identity of a specific mouse/sample. However, genotyping was regularly performed by experimenter.

Reporting for specific materials, systems and methods

We require information from authors about some types of materials, experimental systems and methods used in many studies. Here, indicate whether each material, system or method listed is relevant to your study. If you are not sure if a list item applies to your research, read the appropriate section before selecting a response.

Materials & experimental systems

n/a	Involved in the study	
[ ]	[x]	Antibodies
[ ]	[x]	Eukaryotic cell lines
[x]	[ ]	Palaeontology
[ ]	[x]	Animals and other organisms
[x]	[ ]	Human research participants
[x]	[ ]	Clinical data

Methods

n/a	Involved in the study	
[x]	[ ]	ChIP-seq
[x]	[ ]	Flow cytometry
[x]	[ ]	MRI-based neuroimaging

Antibodies

Antibodies used

Primary antibodies:
UCP1, PA1-24894 (ThermoFisher Scientific, Waltham, MA, USA); Pgc1α, AB 3242 and Actin, MAB1501 (Millipore, Darmstadt, Germany); ASK1, AB45178 (Abcam, Cambridge, UK), pASK1, sc-109911 (Santa Cruz Biotechnology, Dallas, TX, USA), pIRF3, 29047 (Cell Signalling, Danvers, MA, USA), GAPDH, 10494-1-AP (Proteintech, Manchester, UK).

Secondary antibodies:
Goat-anti-rabbit IgG-HRP (ab6721), goat-anti-rat IgG-HRP (ab205720), goat-anti-mouse IgG-HRP (ab6789) (all from Abcam Cambridge, UK; all diluted 1:5000)

Validation

Pgc1α, AB 3242 (Millipore)
Validated for use in Western Blotting. Representative lots detected similar PGC-1α in human, mouse, and rat skeletal muscle tissue, as well as in nuclear and mitochondrial preparations from rat brown adipose tissue (Suwa, M., et al. (2015). J. Sports Sci. Med. 14(3):548-555; Lee, I., et al. (2015). Front. Pharmacol. 6:43; Vincent, G., et al. (2015). Front Physiol. 6:51; Lombardi, A., et al. (2015). PLoS One. 10(2):e0116498; Gouspillou, G., et al. (2014). FASEB J. 28(4):1621-1633; Saleem, A., et al. (2014). Am. J. Physiol. Cell Physiol. 306(3):C241-249).

Actin MAB1501 (Millipore)
Validated for use in Western Blotting. All animal species and cell types with an actin form react by immunoblot with this

antibody.

ASK1, AB45178 (Abcam)
Validated using a knockout cell line. Reacts with mouse and human. Tested for Western Blot application.

pASK1, sc-109911 (Santa Cruz Biotechnology)
Recommended for detection of phosphorylated ASK1 of mouse origin by Western Blotting.
pIRF3 29047 (Cell Signalling)
Reacts with human, mouse and rat. Validated for use in Western Blotting using lysates from IRF-3 knockout mice.

GAPDH, 10494-1-AP (Proteintech).
Tested for use in Western Blotting. Reacts with among others with human, mouse, rat, pig.

Eukaryotic cell lines

Policy information about cell lines

Cell line source(s)	HEK-293LTV cells were obtained from Cell Biolabs (cat# LTV-100).<br>Immortalized white/subcutaneous pre-adipocytes were obtained as previously described (Cell Rep 2016; 16(8):2243-2258).
Authentication	Cell lines used were not authenticated
Mycoplasma contamination	In most but not all cell culture experiments, cells were regularly tested negative for mycoplasma contamination.
Commonly misidentified lines<br>(See ICLAC register)	No commonly misidentified cell lines were used.

Animals and other organisms

Policy information about studies involving animals; ARRIVE guidelines recommended for reporting animal research

Laboratory animals	Mice were generated as described in the Methods section. 6-week-old male mice were put on respective diets. Adipocyte-specific ASK1 knockout and overexpressing mice as well as myeloid-specific ASK1 knockout mice were on a C57BL/6 background. All mice were housed in a specific pathogen-free environment at room temperature (21°C) on a 12-hours-light-dark cycle (light on from 7 am to 7 pm).
Wild animals	Study did not involve wild animals.
Field-collected samples	Study did not involve samples collected in the field.
Ethics oversight	All protocols conformed to the Swiss animal protection laws and were approved by the Cantonal Veterinary Office in Zurich, Switzerland.

Note that full information on the approval of the study protocol must also be provided in the manuscript.
